# Knowledge, Attitude and Factors associated with Self-efficacy in Screening and Treatment of Hepatitis C among Primary Care Doctors in Selangor

**DOI:** 10.51866/oa.454

**Published:** 2024-02-20

**Authors:** Nurulhana Shaikh Mansoor, Farnaza Ariffin, Leny Suzana Suddin, Zati Sabrina Ahmad Zubaidi

**Affiliations:** 1 MBBS, FRCGP[Int], Department of Primary Care Medicine, Faculty of Medicine UiTM, Sungai Buloh Campus, Jalan Hospital, Sungai Buloh, Malaysia. Email: Farnaza@uitm.edu.my; 2 MBBS, MMed Fam Med, Department of Primary Care Medicine, Faculty of Medicine UiTM, Sungai Buloh Campus, Jalan Hospital, Sungai Buloh, Selangor, Malaysia.; 3 MD, MPH, DRPH, Department of Public Health Medicine, Faculty of Medicine UiTM, Sungai Buloh Campus, Jalan Hospital, Sungai Buloh, Selangor, Malaysia.; 4 MBBS, MMed Fam Med., Department of Primary Care Medicine, Faculty of Medicine UiTM, Sungai Buloh Campus, Jalan Hospital, Sungai Buloh, Selangor, Malaysia.

**Keywords:** Hepatitis C, Primary care, Knowledge, Attitude, Self efficacy

## Abstract

**Introduction:**

Primary care doctors (PCDs) play an increasingly important role in the management of hepatitis C. It is essential for PCDs to have good self-efficacy in screening and treating hepatitis C to achieve good outcomes. This study aimed to determine the knowledge and attitude towards and other factors associated with self-efficacy in screening and treating hepatitis C.

**Methods:**

This cross-sectional study was conducted using an online Google Form. PCDs working at primary healthcare clinics were selected via simple random sampling. The online form contained items on sociodemographic and practice characteristics and a validated questionnaire on knowledge, attitude and self-efficacy towards screening and treating hepatitis C. Data were statistically analysed.

**Results:**

A total of 242 PCDs were included in the analysis. The median age was 35 years (interquartile range [IQR]=5). The majority of the PCDs were women (83.9%) and Malay (71.9%) and had a median working experience of 6 years (IQR=6). The mean self-efficacy score was 12.67 (standard deviation=3.38). The factors associated with a higher level of self-efficacy in screening and treating hepatitis C were postgraduate qualification, training within the last one year, better knowledge and attitude scores and prior experience in treating hepatitis C.

**Conclusion:**

The identified factors are crucial in improving the self-efficacy among PCDs in hepatitis C care services. Policymakers are suggested to implement training programmes and encourage continuous medical education, exposure to patient management and postgraduate certification in family medicine to help PCDs in treating hepatitis C better.

## Introduction

In 2022, the Ministry of Health (MoH) Malaysia announced an increase in the availability of point-of-care diagnostic kits and treatment for hepatitis C (hep C) at public primary healthcare clinics.^1^ This pledge sought to improve access to screening, diagnosis and treatment by incorporating hep C management at primary healthcare clinics. It followed a series of initiatives by the MoH to combat the rise in the prevalence of hep C, with 2.5% of patients currently living with the virus and many more who are unaware of their hep C status,^2^ and the increase in the numbers of death due to hep C.^3^ In 2019, the MoH launched the clinical practice guidelines in the management of hep C.^4^ In 2021, the MoH announced the aim for Malaysia to become a hep C treatment hub in offering the latest antiviral treatment in response to the WHO’s mission to reduce new hep C infection by 90%.^5^

The latest antiviral treatment, known as direct-acting antivirals (DAAs), was introduced in 2013. DAAs are more effective, well tolerated and given for 12–24 weeks compared with older injectable interferons that are difficult to administer and associated with severe adverse effects.^6^ Treatment must be initiated among patients with hep C since it can reduce the risk of developing hepatocellular carcinoma.^7^ The diagnostic kit mentioned above is simple and useful in screening hep C virus (HCV). It is a point-of-care serological and nucleic acid test for HCV.^8^ Despite the subsidy and availability of treatment, making hep C essentially curable, the disease remains a public health concern, as the majority of people living with hep C are unaware of their diagnosis, and the disease is usually diagnosed at a later stage with manifestations of liver cirrhosis or hepatocellular carcinoma.^9,10^ Studies show that screening and treating hep C in primary healthcare settings are feasible and as effective as managing the disease in hospital settings.^11^ The inclusion of primary healthcare clinics as the first point of contact for screening and treating hep C plays a major role in the success of this programme. Therefore, the ability and confidence of primary care doctors (PCDs) in fulfilling this task require further scrutiny.

The self-efficacy theory states that it is individuals’ particular set of self-belief or selfconfidence that determines how well they can execute a plan of action in a prospective situation.^12^ Therefore, according to this theory, a higher level of self-efficacy in screening and treating hep C among PCDs will lead to better patient care. This study aimed to determine the knowledge and attitude towards and other factors associated with self-efficacy in hep C screening and treatment among PCDs. The findings of this study are expected to complement the MoH’s vision to improve patient care for hep C.

## Methods

### Study design and population

This cross-sectional study was conducted among PCDs working at public healthcare clinics within the state of Selangor using a self-administered online questionnaire. PCDs comprised family medicine specialists (FMSs) and medical officers. PCDs who were practising at public healthcare clinics in Selangor, were registered with the Malaysian Medical Council and had more than 6 months of experience working at the primary healthcare clinics were included. PCDs who were on long leave of more than 6 months were excluded. Data were collected from June to September 2022.

### Sample size calculation

The sample size was calculated using G*Power version 3.1 for multiple linear regression (MLR) and as previously described by Richmond et al. 13 With an a value of 0.05, a power of 95%, an R^2^ value for hep C knowledge among medical practitioners of 0.32 and an R^2^ value for attitude towards hep C of 0.30, the estimated sample size was 116. Given an attrition rate of 64% for online questionnaires, the link to the questionnaire was distributed to at least 320 PCDs.

### Sampling method and data collection

The PCDs were selected via simple random sampling. There are a total of 79 public healthcare clinics in Selangor, and an updated list of all PCDs working at these clinics was obtained from the district health offices in the state. Simple random sampling was applied to this list using an online random number generator, and the selected PCDs were invited to participate in the study with the help of the FMS in charge of the clinic. The participants were given a link to the online Google Form, which included information regarding the study. Consent was obtained within the online form prior to proceeding to the questions. Reminders were sent 2-4 weeks after the initial invitation.

### Study instrument and validation

The self-administered online questionnaire consisted of items on sociodemographic and practice characteristics as well as knowledge, attitude and self-efficacy towards hep C screening and treatment. The questionnaire was originally designed by Doshi et al. in a United States study, and their findings were published in a peer-reviewed journal.^14^ The questionnaire was developed in English and contains items covering knowledge, attitude and self-rated proficiency (self-efficacy) towards hep C screening and treatment. In this study, it was retained in the original language and unchanged. Permission to use the questionnaire was obtained from the developers.

The sociodemographic and practice characteristics included the participants’ age, sex, ethnicity, educational level, location of practice, type of practice, duration of practice, number of patients seen in an average day, availability of referral services, prior hep C education and estimated number of patients screened, diagnosed and treated for hep C.

The knowledge domain of the questionnaire consists of seven items with pre-determined correct answers. The first item is a standalone question on screening indications. The other six items measure knowledge of hep C screening and treatment and are scored with a minimum sum of 0 and a maximum sum of 6. The attitude domain has 10 items and is scored using a 5-point Likert scale. The score ranges from 1 (strongly disagree) to 5 (strongly agree) for positive statements, while the scoring reverses for negative statements with a maximum score of 50. The self-efficacy domain is scored using a 5-point Likert scale based on five items on aspects of hep C care with a maximum score of 25. For all domains, a higher score represents better knowledge, attitude and self-efficacy.

Content validation, face validation and a pilot study were performed to ensure that the questionnaire was reliable and valid to be used among the Malaysian PCDs. For content validation, a content validation index (CVI) forms were used among six experts from gastroenterology, infectious disease and family medicine, and the calculated S-CVI/Ave for the questionnaire was 0.93. For face validation, the S-CVI/Ave was 0.95. The questionnaire was then pilot-tested on 30 PCDs who were not included in the main study. The Cronbach’s a values for the knowledge, attitude and self-efficacy domains were 0.77, 0.76 and 0.89, respectively. The Cronbach α value for the entire questionnaire was 0.82, indicating that the questionnaire had good internal consistency and reliability.

### Data analysis

The data collected were analysed using SPSS version 28 (IBM, Chicago, IL, USA). The sociodemographic and practice characteristics were evaluated using descriptive statistics. Normally distributed continuous data were presented as means ± standard deviations and non-normally distributed data as medians and interquartile ranges (IQRs). Conversely, categorical data were described as frequencies and percentages. The total mean scores were calculated for the knowledge, attitude and selfefficacy domains. The relationship between the independent variables (sociodemographic and practice characteristics, knowledge and attitude) and the dependent variable (selfefficacy) was determined using simple linear regression. All independent variables with a P-value of <0.25 were included in the MLR using the stepwise method. Statistical significance was considered at a P-value of <0.05. Any significant variables in the MLR were checked for interaction and multicollinearity. The model was then tested for homoscedasticity to evaluate the model fitness. The model was also checked for linearity by generating scatter plots between residuals and predicted values.

## Results

A total of 320 PCDs were invited to participate in this study, among whom 255 agreed, yielding a response rate of 80%. Thirteen PCDs were excluded, as they did not fulfil the inclusion criteria. Thus, a total of 242 participants were included in the final analysis.

Table 1 illustrates the sociodemographic and practice characteristics of the participants. Most participants were women (83.9%) and Malay (71.9%). The median working experience was 6 years (IQR=6). The median age of the participants was 35 years (IQR=5).

**Table 1:** Sociodemographic and practice characteristics (N=242).

Characteristics	Median IQR	n (%)
Sex		
Male		39 (16.1)
Female		203 (83.9)
Age (year)	35 (5)	
Race/ethnicity		
Malay		174 (71.9)
Chinese		16 (6.6)
Indian		48 (19.8)
Others		4 (1.7)
Highest qualification		
Medical degree (e.g. MD or MBBS)		189 (78.1)
Graduate Certificate of Family Medicine or Diploma Family Medicine		38 (15.7)
Postgraduate qualification in family medicine (e.g. Master in Family Medicine, FRACGP or MRCGP [UK])		15 (6.2)
Location of practice		
Urban		204 (84.3)
Rural		38 (15.7)
Clinic with hepatitis C treatment services		
Yes		114 (47.1)
No		111 (45.9)
Unknown		17 (7)
Clinic with a family medicine specialist		234 (96.7)
Yes		8 (3.3)
No		
Duration of practice (year)	6 (6)	
Number of patients seen in an average day	35 (10)	
Percentage of patients at risk of hepatitis C screened		
0%		14 (5.8)
1-20%		171 (70.7)
21-50%		17 (7.0)
51-75%		5 (2.1)
>75%		19 (7.9)
Unknown		16 (6.6)
Number of patients with confirmed hepatitis C		
0		101 (41.7)
1-5		120 (49.6)
6-10		9 (3.7)
11-20		5 (2.1)
>20		7 (2.9)
Number of patients with hepatitis C treated		
0		204 (84.3)
1-10		30 (12.4)
11-20		3 (1.2)
21-30		3 (1.2)
>30		2 (0.8)
Availability of referral services for hepatitis C		
Yes		231 (95.5)
No		3 (1.2)
Unknown		8 (3.3)
Prior hepatitis C training/education within the past 1 year		
Yes		83 (34.3)
No		159 (65.7)

* IQR = Interquartile range

Table 2 shows the median knowledge, mean attitude and mean self-efficacy scores for hep C screening and treatment. A score of 5/6 for knowledge was considered high; a score of 38.11/50 for attitude was considered fairly high; and a score of 12.67/25 for self-efficacy was considered average.

**Table 2:** Knowledge, attitude and self-efficacy scores for hepatitis C screening and treatment.

Domains	Scores
Knowledge (0–6)	5 (3)
Attitude (5–50)	38.11 (5.54)
Self-efficacy (5–25)	12.67 (3.38)

The knowledge score is presented as medians (IQRs) and the attitude and self-efficacy scores as means (SDs).

Table 3 presents the findings of the univariate analysis using simple linear regression. The factors with a P-value of <0.25 in this analysis were included in the MLR analysis.

**Table 3:** Simple linear regression analysis of the factors associated with self-efficacy.

Variables	Simple linear regression	
	b (95% Cl)	P-value
Age	0.23 (0.13,0.33)	<0.001
Sex		
Male	Ref.	Ref.
Female	-0.88 (-2.04, 0.28)	0.135
Qualification		
GCFM	Ref.	Ref.
MBBS	-0.60 (-1.73,0.52)	0.290
Postgraduate qualification infamily medicine	3.97 (2.05, 5-90)	<0.001
Clinic with a family medicine specialist		
No	Ref.	Ref.
Yes	1.73 (0.66,4.11)	0.155
Duration of practice (year)	0.23 (0.11,0.34)	<0.001
Number of patients seen in an average day	-0.03 (-0.05, 0.03)	0.081
Prior hepatitis C training within the past 1 year		
No	Ref.	Ref.
Yes	2.99 (2.18,3.82)	<0.001
Hepatitis C knowledge score	0.78 (0.52, 1.03)	<0.001
Hepatitis C attitude score	0.17(0.10, 0.25)	<0.001
Prior experience in diagnosing hepatitis C	1.22 (0.36,2.07)	0.005
Prior experience in treating hepatitis C	4.26 (3.22,5.31)	<0.001

Dependent variable: Self-efficacy score

Table 4 shows the factors associated with the self-efficacy of the PCDs in hep C screening and treatment. In the MLR analysis, five factors were significantly associated with the self-efficacy score, explaining 37% of the variation (R2=0.37).

**Table 4:** Multiple linear regression analysis of the factors associated with self-efficacy in hepatitis C screening and treatment.

Variables	**Multiple linear regression**	
	b^b^ (95% Cl)	P-value
Qualification GCFM	Ref.	Ref.
MBBS	-0.09 (-1.08,0.89)	0.851
Postgraduate qualification in family medicine	2.25 (0.74, 3.77)	0.004
Prior hepatitis C training within the past 1 year No	Ref.	Ref.
Yes	1.56 (0.75,2.37)	<0.001
Hepatitis C knowledge score	0.33 (0.08,0.057)	0.009
Hepatitis C attitude score	0.08 (0.01,0.14)	0.026
Prior experience in treating hepatitis C No	Ref.	Ref.
Yes	2.72 (1.69,3.76)	<0.001

b Adjusted regression coefficient Stepwise multiple linear regression method was applied. Model assumptions were fulfilled. No multicollinearity was detected. There were no significant interactions between the independent variables. R^2^ = 0.367

## Discussion

The five factors associated with better self-efficacy identified in this study were postgraduate qualification, training in hep C within the last 1 year, better knowledge and attitude scores and previous experience in treating hep C. These factors are essential in shaping future policies to improve hep C management by PCDs at primary healthcare clinics.

The first factor identified was postgraduate qualification. PCDs with postgraduate qualification tend to be more confident in their ability to treat hep C. This could be attributed to them receiving training or having the opportunity to manage patients with hep C in hospitals or during their attachment in primary healthcare clinics. Studies have shown that in general, primary care trainees are more confident about their skills, and there are improvements in knowledge following a vocational training programme.^15^ As part of the healthcare transformation plan, the MoH plans to increase the number of FMSs in Malaysia. The aim is to have about 8000 FMSs to be placed in healthcare clinics nationwide.^16^ Currently, in Malaysia, doctors are not required to undergo postgraduate training following a basic medical degree to practise in a primary care setting. The findings of this study support the need to introduce family medicine postgraduate certification as a requirement for entry into the field of family medicine. Strategies should be implemented to encourage PCDs to pursue postgraduate study and become specialists. This will contribute to the production of competent and skilled doctors to cater to the current health needs including the management of hep C.

In the present study, better self-efficacy in hep C management was noted in the PCDs who received prior training or education in hep C management within the past 1 year. Such training can be in the form of seminars, continuing medical education (CME) or courses. Training and CME are necessary for PCDs to stay updated with new DAAs and strengthen their knowledge in managing hep C effectively.^17^ This study also found that a small increase in the knowledge and attitude scores increased the self-efficacy score. This finding is consistent with the self-efficacy theory, wherein knowledge and attitude are contributors to performance accomplishment. With more knowledge and better attitude, individuals are more likely to feel able to accomplish a specific task.^18^ Insufficient knowledge among PCDs is one of the barriers to scaling up hep C treatment at primary healthcare clinics in Malaysia.^19^ PCDs’ knowledge, belief and feel towards hep C play an important role in hep C care provision.^13^ It is proven that educational intervention and training improve knowledge and attitude.^20^ The Malaysian clinical practice guidelines in hep C management should be made accessible to all PCDs, and echo training can be conducted to improve knowledge, attitude and self-efficacy.

Herein, the descriptive analysis revealed that the PCDs were good at identifying patients at a high risk for hep C, such as those with HIV infection and IV drug users. However, they were less aware of the risk among patients who had blood transfusion prior to 1994. A targeted training can improve the knowledge and attitude towards hep C and self-efficacy to identify patients at risk, conduct point-of-care testing, initiate DAA treatment and perform necessary referrals to hospitals.^20^ Enhancing hep C training and using effective teaching methods tailored towards PCDs can improve knowledge and attitude, leading to better selfefficacy and hep C outcomes.^20^ It is known that improving PCDs’ capacity to screen and treat will be most impactful if the focus is on training those who are exposed to patients with HCV and who have an existing supportive system.^21^

Finally, the PCDs with prior experience in managing hep C showed a higher level of self-efficacy in this study. This finding is supported by the self-efficacy theory in that past experience is the most crucial source of self-efficacy.^12^ People are more likely to feel competent and perform a task well if they have previously completed a similar task.^12^ Hep C testing and treatment within primary healthcare settings in Malaysia were introduced in 2018,^9^ providing a wide range of hep C care services, including the initiation of DAAs, while hospitals focused on treating patients with complications such as cirrhosis and treatment failure.^19^ Despite this, there are PCDs still lacking experience in managing hep C, which is associated with the lack of knowledge and confidence in managing such.^19^ Not all PCDs are placed in hep C clinics, and only some will have the opportunity to manage patients with hep C. It is important for rota-makers to ensure that PCDs have the opportunity and exposure to manage hep C when they are attached at primary healthcare clinics. This is because PCDs’ capacity in screening and treating hep C is most impactful among those who have handled large numbers of hep C cases and received good training.^14,21^

The five factors identified in the study are important in improving the self-efficacy among PCDs in hep C care services. Policymakers are suggested to implement training programmes and encourage CME, exposure to patient management and postgraduate certification in family medicine to help PCDs in treating hep C better in Malaysia.

To the authors’ knowledge, this study is the first local study to evaluate the knowledge and attitude towards and other factors associated with self-efficacy in hep C screening and treatment among PCDs. The findings could guide policymakers in implementing measures to improve PCDs’ self-efficacy in managing hep C. Nonetheless, there are limitations that should be considered. One limitation is that the data were collected only in one state (Selangor) and may therefore not represent the entire country. Another limitation is the use of a self-reported online questionnaire, which may yield recall bias.

## Data sharing statement

Data are available upon request.

**How does this paper make a difference in general practice?**

- Primary care doctors have average knowledge, attitude and self-efficacy towards managing hepatitis C. Their knowledge, attitude and self-efficacy must be enhanced to achieve better outcomes.
- The findings emphasise the importance of postgraduate qualification and training, as these factors are associated with better self-efficacy in managing hepatitis C.
- The findings could help policymakers in providing training and exposure to primary care doctors to improve their self-efficacy, knowledge and attitude towards hep C management.
